# Machine Learning-Identified Potent Antimicrobial Peptides Against Multidrug-Resistant Bacteria and Skin Infections

**DOI:** 10.3390/antibiotics14111172

**Published:** 2025-11-20

**Authors:** Gizem Babuççu, Nikitha Vavilthota, Colin Bournez, Leonie de Boer, Robert A. Cordfunke, Peter H. Nibbering, Gerard J. P. van Westen, Jan W. Drijfhout, Sebastian A. J. Zaat, Martijn Riool

**Affiliations:** 1 Department of Medical Microbiology and Infection Prevention, Amsterdam Institute for Infection and Immunity, Amsterdam University Medical Centre, University of Amsterdam, 1105 AZ Amsterdam, The Netherlands; g.babuccu@amsterdamumc.nl (G.B.); n.vavilthota@amsterdamumc.nl (N.V.); l.deboer@amsterdamumc.nl (L.d.B.); 2 Computational Drug Discovery, Medicinal Chemistry, Leiden Academic Center for Drug Research, Leiden University, 2300 RA Leiden, The Netherlandsgerard@lacdr.leidenuniv.nl (G.J.P.v.W.); 3 Department of Immunology, Leiden University Medical Center, 2300 RC Leiden, The Netherlands; r.a.cordfunke@lumc.nl (R.A.C.);; 4 Department of Infectious Diseases, Leiden University Medical Center, 2300 RC Leiden, The Netherlands; 5 Laboratory for Experimental Trauma Surgery, Department of Trauma Surgery, University Hospital Regensburg, 93053 Regensburg, Germany

**Keywords:** antimicrobial peptides (AMPs), machine learning (ML), antimicrobial resistance, biofilm eradication, wound infection, 3D human epidermal model

## Abstract

Background: The escalating global crisis of antibiotic resistance necessitates the discovery of novel antimicrobial agents. Antimicrobial peptides (AMPs) represent a promising alternative to combat multidrug-resistant (MDR) pathogens. Because traditional AMP discovery is labour-intensive and costly, machine learning (ML) is applied to identify AMPs effective against MDR bacteria and skin infections. Methods: The ML-based CalcAMP model predicts the antimicrobial activity of 16,384 unique 14-amino-acid peptide sequences, resulting in a novel Guided Designed Smart antimicrobial Therapeutic (GDST) peptide catalogue. Parent sequences and retro-inverso (RI) variants of two prime GDST peptides undergo extensive testing against MDR bacteria and in skin infection models. Results: GDST-038 and GDST-045, along with their RI variants, show potent antimicrobial activity against *Acinetobacter baumannii* and *Staphylococcus aureus*, rapidly depolarizing the cytoplasmic membrane, exhibiting broad-spectrum bactericidal effects against ESKAPE pathogens, and causing minimal haemolysis. RI variants display superior *A. baumannii* biofilm killing compared to parent sequences, while all GDST peptides achieve >3-log reductions in *S. aureus* biofilm CFU within 24 h. Potent efficacy is observed in a 3D human skin epidermal infection model, with elimination of *S. aureus* at ≥15 μM. No resistance develops after 22 passages. Conclusions: ML-driven screening enables rapid identification of two novel candidate AMPs, highlighting the therapeutic potential of GDST peptides for MDR bacterial infections.

## 1. Introduction

Chronic wounds frequently harbour antibiotic-resistant bacteria, including those resistant to last-resort antibiotics like third-generation cephalosporins, carbapenems, and polymyxins [1,2]. The most common chronic wounds include vascular ulcers, diabetic ulcers, and pressure ulcers, primarily affecting the lower limbs [3]. Opportunistic pathogens such as *Acinetobacter baumannii* and *Staphylococcus aureus* often colonize skin wounds and form biofilms, reducing antibiotic efficacy, impairing immune defences, and complicating infection eradication [4,5]. The growing global challenge of antimicrobial resistance (AMR) has intensified the need for effective treatments, underscoring the urgency of developing novel, broad-spectrum, and sustainable therapeutic strategies [6].

Antimicrobial peptides (AMPs) are small, typically positively charged, amphipathic molecules that form an effective first line of defence against pathogenic microorganisms in the host’s innate immune system [7]. Their amphipathic structure enables them to disrupt microbial cell membranes, causing loss of membrane integrity [8,9]. AMPs possess broad-spectrum activity against bacteria, fungi, viruses, parasites, and even cancer cells, further broadening their therapeutic potential [10,11,12,13]. Beyond antimicrobial activity, some peptides also promote wound healing, immune cell activation, and angiogenesis [14,15,16]. Despite their promise, discovering AMPs through traditional methods remains costly and time-consuming, relying on high-throughput screening across multiple bacterial strains. This situation has become critical due to the rise of antibiotic-resistant strains and the limited availability of new, effective antibiotics, placing immense pressure on healthcare systems.

To address these problems, there is increasing interest in incorporating state-of-the-art computational approaches, such as machine learning (ML), to accelerate drug discovery and optimize compounds with predefined physicochemical and biological properties [17,18,19,20,21]. Because experimental conditions and organisms differ between studies and screens, data from these sources may be non-standard. Generating standardized datasets, such as through in-house library screens, will be critical for ensuring data quality, which is required for accurate ML model training and benchmarking. In response to the increasing threat of AMR, we recently developed CalcAMP, an in silico ML-based model that predicts AMP activity against both Gram-negative and Gram-positive bacteria with an accuracy of 81%, among the highest reported for current ML models [22]. CalcAMP enables integration of computational modelling with accurate predictions of peptide antimicrobial activity, thereby accelerating the discovery of novel AMPs with therapeutic potential.

In this study, we developed and evaluated a novel collection of ‘Guided Designed Smart antimicrobial Therapeutics’ (GDST) peptides using CalcAMP’s ML prediction capabilities. We selected two prime candidate AMPs and included their retro-inverso (RI) variants, which are reported to evade protease recognition and thus exhibit marked resistance to proteolytic degradation [23]. We assessed their broad bactericidal activity, including activity against the ESKAPE panel of multidrug-resistant (MDR) bacteria [24], cytotoxicity, propensity for bacteria to develop exposure-induced resistance, and biofilm eradication efficacy against *A. baumannii* and *S. aureus*. Finally, we evaluated their efficacy in a 3D human epidermal skin equivalent infection model to closely mimic patient conditions.

## 2. Results

### 2.1. Selection of CalcAMP-Predicted Antimicrobial Peptides

To identify the most promising AMPs, we used CalcAMP to predict the antimicrobial activity of peptides within our design space of 16,384 peptides. Alanine scanning identified that no particular amino acid position within the sequence was essential for bactericidal activity against either *A. baumannii* or *S. aureus* (Table 1). All 14 alanine-substituted peptides showed similar antimicrobial activity against *A. baumannii* and *S. aureus* in RPMI, with cidal concentrations of ≤1–10 μM. The addition of 50% plasma only slightly affected activity against *A. baumannii* but significantly reduced activity against *S. aureus*. All peptides showed haemolytic activity at 100 μM.

The top 14 predicted sequences, ranked by predicted activity against Gram-negative bacteria, had prediction values ranging from 0.912 to 0.890 (Table 2). These 14 peptides exhibited activity comparable to alanine-scan peptides against *A. baumannii* and *S. aureus* in RPMI (LC99.9_18h_: ≤1–10 μM). In 50% plasma, activity against *A. baumannii* showed larger variability among the peptides (≤1–100 μM), while activity against *S. aureus* in plasma was detected at 10 or 100 μM, with haemolytic activity at 100 μM.

The two best-performing GDST peptides were selected based on three criteria: (i) activity against both *A. baumannii* and *S. aureus* in RPMI, (ii) retention of activity in 50% plasma, and (iii) minimal haemolytic activity. GDST-038 and GDST-045 emerged as the two most active peptides, achieving LC99.9_18h_ values of ≤1 μM against both species in RPMI, ≤1 μM against *A. baumannii* in 50% plasma, and 10 μM against *S. aureus* in 50% plasma (Table 2), with haemolytic activity only at 100 µM.

Peptides predicted to have low antimicrobial activity (prediction values of 0.264–0.492) displayed minimal activity against both bacterial species (LC99.9_18h_: 100 or ≥100 µM) and showed no activity in 50% plasma, not even at 100 µM (Table S1). These peptides also exhibited low haemolytic activity at concentrations above 100 μM (i.e., higher than the highest concentration tested).

### 2.2. Bactericidal Activity and Cytotoxicity of GDST-038 and GDST-045

The bactericidal activity and cytotoxicity of GDST-038 and GDST-045 were evaluated against MDR *A. baumannii* and *S. aureus* JAR060131, and compared to reference peptides LL-37, bactericidal peptide 2 (BP2), pexiganan, SAAP-148, and thrombocidin (TC) 84 (Table S2).

Within 18 h in RPMI, reference peptides achieved ≥99.9% killing of *A. baumannii* and *S. aureus* at concentrations of 0.23–7.5 μM, except LL-37, which showed no activity against *S. aureus* even at the highest tested concentration (120 μM). GDST-038, GDST-038-RI, GDST-045, and GDST-045-RI achieved the same level of killing at 0.47–3.75 μM (Table 3). In 50% plasma, BP2, pexiganan, SAAP-148, and TC-84 retained activity against both pathogens (LC99.9_18h_: 0.47–7.5 µM), whereas LL-37 exhibited markedly reduced activity against *A. baumannii* compared to RPMI (1.88 vs. 60 µM). Notably, both parent GDST peptides and their RI variants maintained high activity in 50% plasma, with LC99.9_18h_ values of 0.47–3.75 µM. Furthermore, GDST peptides achieved ≥99.9% killing of *A. baumannii* and *S. aureus* within just 2 h at concentrations of 0.94–3.75 μM in both RPMI and 50% plasma, demonstrating comparable or superior efficacy to the reference peptides (LC99.9_2h_: 0.23–>120 µM).

Cytotoxicity of GDST peptides was observed at 3.75 µM, within the range of the reference peptides (0.47–15 µM). In contrast, LL-37 exhibited cytotoxicity at 7.5–15 µM but required much higher concentrations (>120 µM) to kill *S. aureus* in RPMI, even after 18 h of incubation (Figure S1).

GDST-038 and GDST-038-RI were active against MDR ESKAPE pathogens (Table 4) within 2 h at concentrations of 0.94–15 µM in RPMI and in 50% plasma, except for *E. cloacae, S. aureus,* and *P. aeruginosa* in 50% plasma, which required 30–60 µM. Both peptides retained activity at 18 h (1.88–15 µM). GDST-045 and GDST-045-RI killed ESKAPE pathogens at 0.94–15 µM in RPMI and at 0.94–120 µM in 50% plasma within 2 h, improving to 0.94–30 μM after 18 h. SAAP-148 showed an 8 to 16-fold reduction in activity in plasma after 18 h, except for *E. cloacae*, which was eradicated at 4-fold lower concentrations in plasma. Against *A. baumannii* and *K. pneumoniae*, SAAP-148 showed similar activity in both RPMI and 50% plasma.

### 2.3. Time–Kill Kinetics of GDST Peptides Against A. baumannii and S. aureus

**Figure 1:**
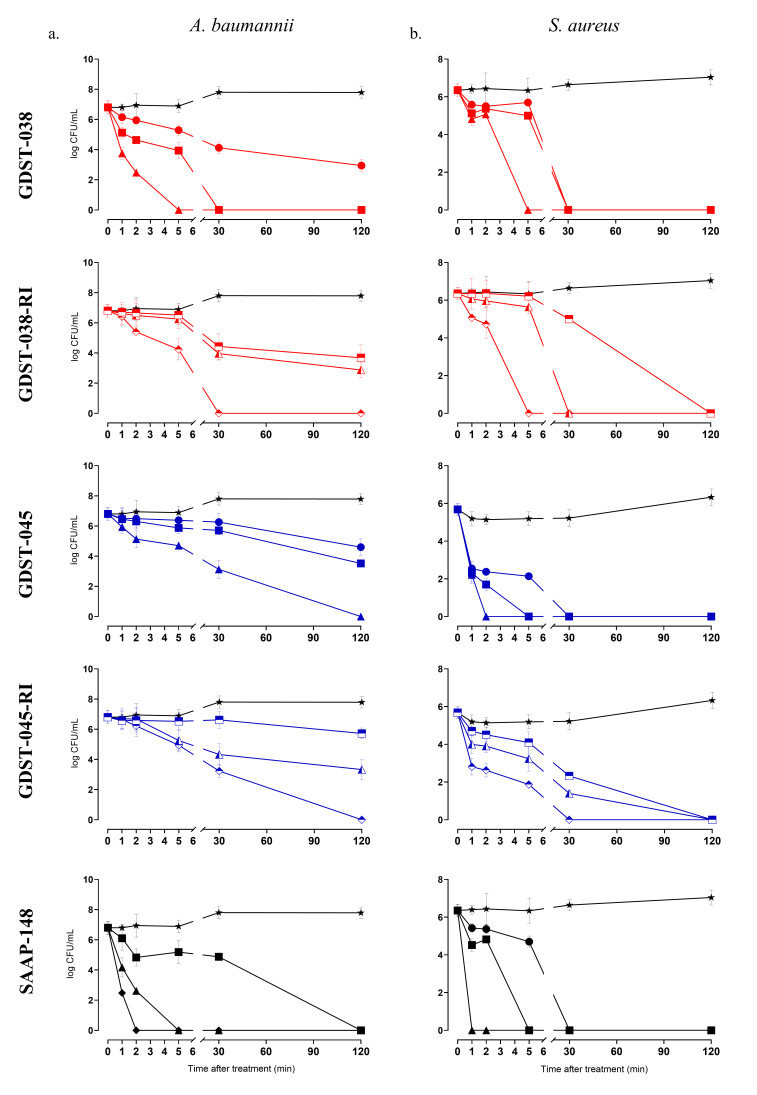
Time–kill kinetics of *A. baumannii* RUH875 (**a**) and *S. aureus* JAR060131 (**b**) exposed to GDST-038 and GDST-038-RI (red symbols), GDST-045 and GDST-045-RI (blue symbols),and SAAP-148 at concentrations of 0- (star; no peptide, control), 0.5-, 1-, and 2-fold LC99.9_2h_ in RPMI for 1 to 120 min (see Table 4). Concentrations depicted as circle: 0.47 μM, square: 0.94 μM, upward triangle: 1.88 μM, and diamond: 3.75 μM. Open symbols represent RI GDST peptide variants. Experiments were conducted in duplicate and repeated three times.

The time–kill kinetics of GDST-038, GDST-045, and their RI variants were evaluated in comparison to SAAP-148. GDST-038 and GDST-045 achieved >90% killing of *A. baumannii* (Figure 1a) and *S. aureus* (Figure 1b) within 5 min at 1.88 μM. Additionally, ≥3.75 μM of GDST-038-RI killed over 90% of both *A. baumannii* and *S. aureus* within 5 min. Complete killing of *S. aureus* was observed within 2 min, while *A. baumannii* required exposure for 120 min at this concentration. GDST-045-RI achieved total eradication of *A. baumannii* and *S. aureus* within 120 and 30 min, respectively, at 3.75 μM.

Overall, GDST-038 achieved complete killing of both species within 5 min at 1.88 μM, while GDST-045 eradicated *S. aureus* within 2 min at the same concentration, suggesting a rapid bactericidal mechanism similar to that of SAAP-148 [24]. In contrast, the RI variants of GDST-038 and GDST-045 exhibited slower killing against *A. baumannii* as compared to their parent sequences.

### 2.4. Membrane Permeabilization of A. baumannii and S. aureus by GDST Peptides

**Figure 2:**
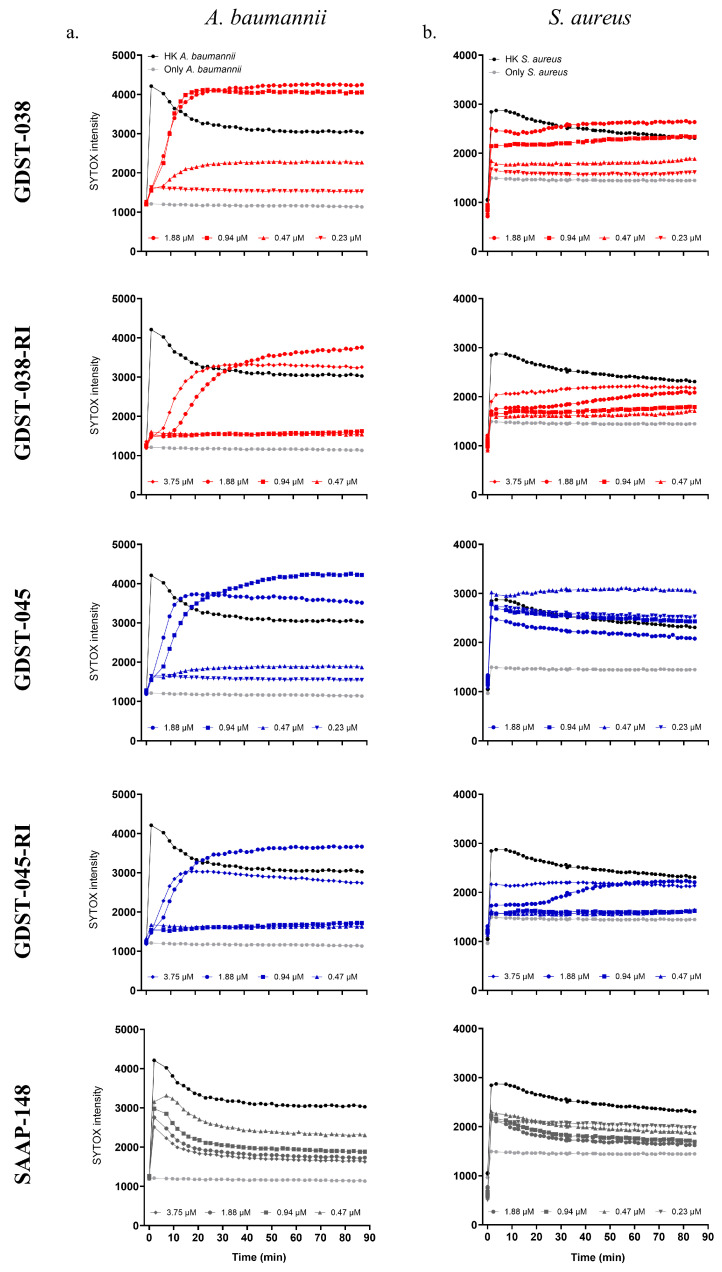
Membrane permeabilization analysis of *A. baumannii* (**a**), and *S. aureus* (**b**) treated with GDST-038, GDST-038-RI, GDST-045, GDST-045-RI, and SAAP-148. GDST-038 and GDST-038-RI are shown in red symbols, and GDST-045, GDST-045-RI in blue. Heat-killed (HK) bacteria (black line) represent maximum membrane permeabilization, while untreated bacteria (light grey line) serve as the intact-cell control.

To investigate the mechanism of action of GDST peptides, membrane integrity was assessed using SYTOX Green uptake. GDST and GDST-RI peptides permeabilized the cytoplasmic membrane of *A. baumannii* and *S*. *aureus* in a predominantly concentration-dependent manner (Figure 2).

The reference peptide SAAP-148, a membrane-disrupting cationic peptide [24], induced measurable membrane permeabilization at all tested concentrations (0.47–3.75 µM). A gradual increase in SYTOX fluorescence intensity was detected after the addition of GDST-038, GDST-038-RI, GDST-045, or GDST-045-RI to *A. baumannii* at 1- and 2-fold the LC99.9_2h_ concentrations, with fluorescence levels similar to heat-killed controls over 90 min (Figure 2a). GDST-038 also increased SYTOX uptake at sublethal concentrations (0.47 µM) compared to untreated bacteria. All GDST peptides, their RI variants, and SAAP-148 caused a concentration-dependent increase in membrane permeabilization over 90 min.

Against *S. aureus*, GDST-038 and GDST-045 caused an immediate rise in fluorescence intensity within 1 min of peptide addition at both sublethal and lethal concentrations, which remained stable over 90 min (Figure 2b). GDST-038-RI and GDST-045-RI showed similar activity at 1.88–3.75 µM, while SAAP-148 induced a rapid fluorescence increase at 0.23–1.88 µM, followed by a slight decline, as also seen with heat-killed controls.

These findings indicate that GDST peptides and their RI variants induce rapid and concentration-dependent membrane depolarization in both *A. baumannii* and *S. aureus,* with almost instant full permeabilization of the *S. aureus* cells.

### 2.5. Resistance Evolution to GDST Peptides

**Figure 3:**
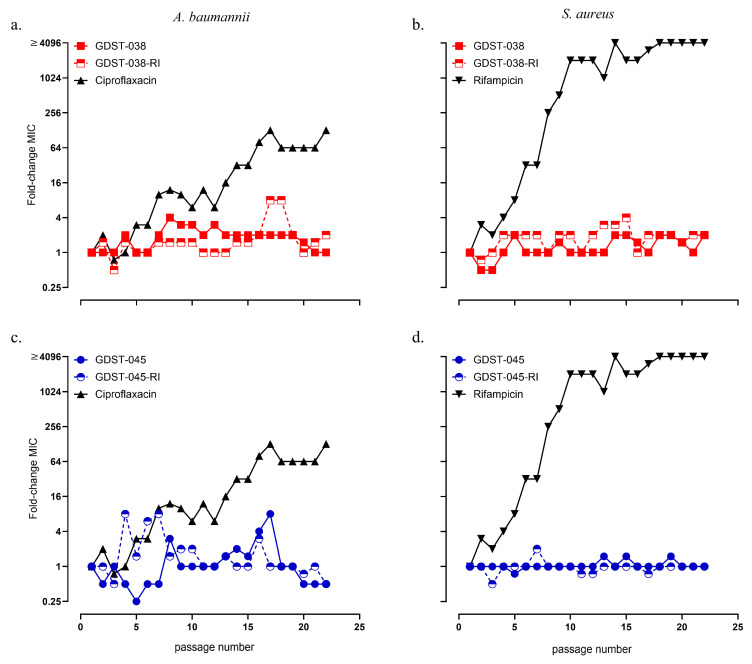
Resistance evolution in *A. baumannii* RUH875 (**a**,**c**) and *S. aureus* JAR060131 (**b**,**d**) to GDST-038, GDST-045, and their RI variants, compared to ciprofloxacin and rifampicin controls. Data represent fold changes in minimal inhibitory concentration (MIC) relative to the MIC at the first passage. Values indicate medians of four replicates. Open symbols represent RI GDST peptide variants.

To evaluate the potential for resistance development, *A. baumannii* and *S. aureus* were serially passaged in sub-inhibitory concentrations of the GDST peptides. No significant increase in MIC was observed in *A. baumannii* exposed to sub-inhibitory concentrations of GDST-038 and GDST-038-RI over 22 passages (Figure 3a). The MIC for GDST-038 remained stable at 0.47 µM, while GDST-038-RI showed only a slight increase, from 0.94 µM at the 1st passage to 1.88 µM by the 22nd passage. Similarly, *A. baumannii* exposed to GDST-045 and GDST-045-RI exhibited no substantial MIC elevation over the 22 passages (Figure 3c).

The MIC for GDST-045 was 0.94 µM at the 1st passage, peaked at 7.50 µM by passage 16, but returned to 0.47 µM by passage 22. For GDST-045-RI, the MIC initially remained steady at 0.94 µM and decreased to 0.47 µM by the final passage. In contrast, ciprofloxacin exposure led to a pronounced MIC increase in *A. baumannii* starting at passage 8, reaching a 128-fold increase after 22 passages (from 0.5 to 64 µg/mL).

Likewise, no resistance development was observed in *S. aureus* exposed to GDST-038, GDST-045, or their RI variants. The MIC of GDST-038 and GDST-038-RI increased only slightly from 0.94 µM at the 1st passage to 1.88 µM at the 22nd passage (Figure 3b). The MIC for both GDST-045 and GDST-045-RI remained constant at 0.47 µM throughout all passages (Figure 3d). In stark contrast, exposure to rifampicin resulted in a rapid and dramatic MIC increase in *S. aureus*, with a >4096-fold rise—from 0.25 to 4096 µg/mL—after 22 passages.

### 2.6. Biofilm-Killing Capacity of GDST Peptides

To assess the biofilm-killing capacity of the GDST peptides, 24 h *A. baumannii* and *S. aureus* formed biofilms were treated with GDST-038, GDST-045, their RI variants, or the reference peptides SAAP-148, TC84, BP2, pexiganan, and LL-37, for 2 h and 24 h.

**Figure 4:**
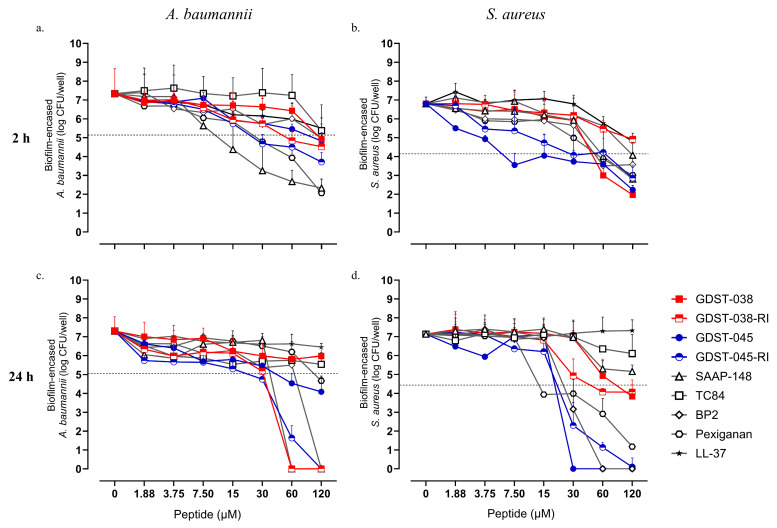
Biofilm killing activity of GDST-038, GDST-045, their RI variants, and reference peptides against 24 h established biofilms of *A. baumannii* RUH875 and *S. aureus* JAR060131. Viable bacteria (log_10_ CFU) were measured after 2 h (**a**,**b**) and 24 h (**c**,**d**) of peptide treatment. Data are median values from three independent experiments, each with four to six technical replicates. The dashed line represents a 3-log CFU reduction relative to the number of CFUs in the biofilms at the start of the exposure to the peptides, which was 8.13 log for *A. baumannii* biofilm 2 h treatment; 8.06 log for *A. baumannii* biofilm 24 h treatment; 7.14 log for *S. aureus* biofilm 2 h treatment; and 7.43 log for *S. aureus* biofilm 24 h treatment.

Within 2 h, GDST-038 at 120 µM, GDST-038-RI at 60 µM, and GDST-045 at 60 µM killed ≥3 log CFU of *A. baumannii* compared to the untreated biofilm control (*p* < 0.001) (Figure 4a). GDST-045-RI achieved more than 3-log *A. baumannii* killing at ≥30 µM (*p* < 0.001). Among the reference peptides, SAAP-148 and pexiganan achieved 3-log killing of *A. baumannii* biofilm at concentrations of ≥15 µM and ≥30 µM, respectively. At 120 µM, GDST-045-RI exhibited a significantly greater biofilm-killing activity than the reference peptides LL-37 and TC-84 (*p* < 0.05).

Following 24 h treatment, complete eradication of *A. baumannii* biofilm was observed with ≥60 µM of GDST-038-RI and 120 µM of GDST-045-RI (both *p* < 0.001) (Figure 4c). GDST-045 achieved a 3.5-log reduction in *A. baumannii* biofilm CFU at ≥60 µM (*p* < 0.01), while GDST-038 did not cause significant biofilm CFU reduction. In contrast, the RI variants achieved complete eradication of *A. baumannii* biofilm, similar to SAAP-148 and BP2. GDST-038-RI and GDST-045-RI also displayed significantly greater biofilm-killing effects than LL-37, pexiganan, and TC84 at the highest concentration tested (120 µM; *p* < 0.001). Interestingly, the >3-log reduction in biofilm CFU observed with pexiganan after 2 h was not sustained after 24 h, and neither TC84 nor LL-37 achieved significant biofilm killing of *A. baumannii* at concentrations up to 120 µM at any treatment time.

*S. aureus* biofilms were also treated with the GDST peptides, their RI variants, and the reference peptides. Within 2 h, GDST-038 at ≥60 µM; GDST-045 at ≥7.5 µM, and GDST-045-RI at ≥30 µM resulted in ≥3-log reduction in numbers of biofilm CFU of *S. aureus* (*p* < 0.001) (Figure 4b). Notably, GDST-038 achieved a 5.1-log CFU reduction, significantly outperforming all reference peptides tested (*p* < 0.05), although its RI variant was less effective. GDST-045 exhibited superior *S. aureus* biofilm-killing activity compared to the reference peptides SAAP-148, TC84, BP2, LL-37, and pexiganan within the same timeframe (*p* < 0.01).

Within 24 h, complete eradication of *S. aureus* biofilm was achieved with GDST-045 at ≥30 µM and GDST-045-RI at 120 µM, alongside BP2 at ≥60 µM (*p* < 0.001) (Figure 4d). Additionally, GDST-038 at 120 µM and GDST-038-RI at ≥60 µM achieved ≥3-log reductions in *S. aureus* biofilm CFU (*p* < 0.001). Among the reference peptides, BP2 and pexiganan demonstrated comparable activity, achieving >3 log killing of *S. aureus* at ≥30 µM (*p* < 0.001), while TC84 and LL-37 did not exhibit significant biofilm-killing effects. Overall, while both parent and RI variants of GDST peptides effectively eradicated *S. aureus* biofilms, the RI peptides showed superior activity against *A. baumannii* compared to their parent counterparts, GDST-038 and GDST-045.

### 2.7. GDST Peptides Effectively Eradicate Non-Adherent and Adherent S. aureus in a 3D Human Skin Equivalent Infection Model

The efficacy of GDST-038, GDST-045, and their RI variants was evaluated in a 3D human skin equivalent infection model colonized with *S. aureus* (Figure S2). Numbers of both non-adherent and adherent bacteria were quantified.

**Figure 5:**
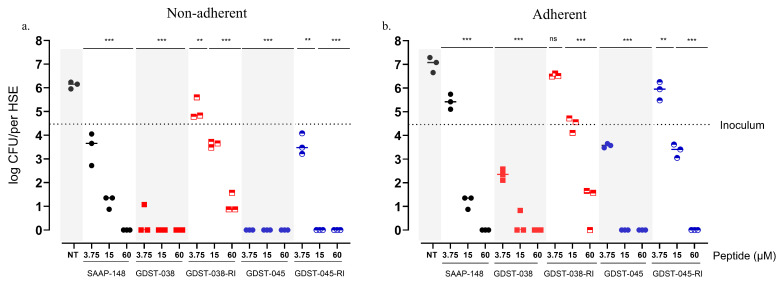
Bactericidal activity of GDST-038, GDST-045, their RI-variants, and SAAP-148 against *S. aureus* JAR060131 infection in a human skin equivalent (HSE) model. Bacterial loads (log CFU/HSE) in the non-adherent (**a**) and the adherent (**b**) fractions of the 3D epidermal models after 4 h of peptide exposure. Individual data points represent independent experiments. NT indicates no-treatment control (PBS only). Statistical analysis: one-way ANOVA with Dunnett’s post-hoc test comparing treated groups to NT control. Significance indicated by ** *p* < 0.001; *** *p* < 0.001; ns: nonsignificant compared to control.

In the planktonic (non-adherent) fraction, GDST-038 and GDST-045 demonstrated superior bactericidal activity compared to their RI variants and the reference peptide SAAP-148 (Figure 5a). Non-treated control (NT) samples showed a high bacterial load of 6.24 log CFU per HSE. SAAP-148 treatment reduced the bacterial burden to below the detection limit at 60 µM, with a 3-log CFU reduction observed at 15 µM. GDST-038 and GDST-045 completely eradicated non-adherent *S. aureus* at all tested concentrations (3.75 µM, 15 µM, and 60 µM; all *p* < 0.001). GDST-038-RI and GDST-045-RI exhibited slightly lower efficacy but still achieved significant bacterial reductions at 15 µM and 60 µM (both *p* < 0.001).

Higher peptide concentrations were required to eradicate adherent *S. aureus* (Figure 5b), NT samples showed bacterial growth during the 4 h treatment, resulting in a bacterial load of approximately log 7 CFU per HSE. SAAP-148 eradicated bacteria only at 60 µM, whereas GDST-038 and GDST-045 exhibited superior efficacy, completely eradicating adherent bacteria at 15 µM (both *p* < 0.001). GDST-038-RI and GDST-045-RI showed concentration-dependent activity and significant reductions in adherent *S. aureus* at 60 µM and 15 µM, respectively (both *p* < 0.001).

Overall, among all tested peptides, GDST-038 and GDST-045 demonstrated the most potent activity against both adherent and non-adherent *S. aureus*, achieving complete eradication at lower concentrations than the RI variants and SAAP-148. These findings highlight their potential in eradicating *S. aureus* in a complex human skin-mimicking environment.

## 3. Discussion

Antibiotics have saved millions of lives, yet the rise of MDR Gram-negative and Gram-positive bacteria poses serious threats to treatment efficacy [25]. Contributing to a solution, recent advancements in ML have opened new avenues for drug discovery, including the identification of novel AMPs [26,27,28]. In this study, we employed the ML tool CalcAMP, which has demonstrated high predictive accuracy, to preselect promising “GDST” peptides from a library of 16,384 peptide sequences. Using this approach, we successfully identified candidates for which the predicted activity was confirmed in extensive wet lab analysis. The CalcAMP-predicted peptides GDST-038 and GDST-045, along with their D-amino acid RI variants, were identified as potent candidates for the treatment of biofilms and human skin infection based on their good performance in HSE infection models. Peptides GDST-038 and GDST-045, and their RI variants, are highly effective against Gram-negative and Gram-positive wound pathogens, with efficacy comparable to or superior to established AMPs such as LL-37 [29], BP2 [30], pexiganan [31], SAAP-148 [24], and TC84 [32]. Notably, GDST peptides and their RI variants retained strong antimicrobial effectiveness in physiological environments, including 50% plasma, which closely mimics wound fluid. They were also very effective against MDR ESKAPE pathogens. GDST peptides and RI variants exerted rapid membrane permeabilization and killing of planktonic bacteria within minutes. Their RI variants outperformed the parent peptides against *A. baumannii* biofilms, where AMP activity is often compromised by proteases [33]. All GDST parent peptides effectively eradicated *S. aureus* biofilms. In a 3D HSE model, GDST peptides eradicated *S. aureus* infection. Thus, the CalcAMP tool allowed the identification of highly relevant novel AMPs against biofilms and skin infection.

Whereas conventional antibiotics primarily eliminate bacteria by interfering with metabolism-dependent processes, AMPs typically target bacterial cell membranes. Since transferable changes in bacterial cell membrane composition occur only infrequently, transferable bacterial resistance to AMPs is considered unlikely, although resistance to AMPs in vitro has been reported [34]. The ability of GDST peptides and RI variants to rapidly permeabilize bacterial membranes and kill planktonic cells within minutes suggests that such swift, non-specific membrane disruption leaves limited opportunity for adaptive resistance mechanisms to emerge. Notably, the peptides permeabilized *S. aureus* membranes more rapidly than those of *A. baumannii*, possibly due to the additional barrier membrane present in Gram-negative bacteria. In addition to their primary membrane-targeting activity, it is possible that GDST peptides will interact with specific intracellular targets once internalized, increasing their overall bactericidal potency. For example, transient pore formation may allow GDST peptides to enter the cytoplasm and interfere with essential processes such as DNA replication, ribosomal function, or enzyme activity. Importantly, both parent and RI variants of the GDST peptides showed no induction of resistance development over 22 passages at sub-inhibitory concentrations, whereas resistance to ciprofloxacin and rifampicin rapidly developed in *A. baumannii* and *S. aureus*, respectively. This combination of a membrane-targeting mode of action and the absence of resistance development highlights the potential of our GDST peptides for future clinical applications.

A broad and favourable therapeutic window is a key advantage for clinically viable AMPs [35], and our lead GDST peptides show promising characteristics in this regard. Initial screening identified GDST-038 and GDST-045 as having among the lowest haemolytic activity of all GDST peptides tested. Subsequent cytotoxicity assessment in human fibroblast cells revealed that GDST peptides were less cytotoxic than SAAP-148, which, like the GDST peptides, has been derived from LL-37 [36,37]. Importantly, no cell damage was observed in the 3D HSE models treated with GDST peptides, supporting their potential safety in more physiologically relevant systems. Further studies, including a more detailed evaluation of cytotoxicity in 3D skin models and antimicrobial activity in in vivo infection models, will be required to progress towards possible translation. Further reduction of off-target effects will be important for their intended application. Recent advancements in functionalized peptide delivery, such as polymer systems [38,39] and 3D printed platforms [40], offer possibilities for enhancing AMP therapeutic profiles. Other advanced formulations, such as nanocarriers, have shown success in AMP delivery, as demonstrated with LL-37 and its derivatives [41,42,43], and could similarly benefit the GDST peptides.

Addressing biofilm-related infections is a considerable challenge in modern healthcare. Antibiotics often fail to eliminate bacterial biofilms, whereas AMPs can penetrate and disrupt biofilms by first engaging in electrostatic interactions via their net positive charge, followed by hydrophobic interactions that facilitate deeper penetration [44], allowing them to kill even dormant bacteria and persister cells [24]. GDST-038, GDST-045, and their RI variants displayed potent *A. baumannii* and *S. aureus* biofilm-eradication capabilities within 2 h of treatment and were superior to reference peptides TC84, LL-37, and pexiganan. Notably, the RI variants of both GDST-038 and GDST-045 demonstrated superior efficacy against MDR *A. baumannii* biofilms compared to parent sequences, particularly in 24 h treatment. Since the normal and RI variant peptides in planktonic testing do not differ much in activity, this difference in late efficacy suggests that the RI variants remain active for a longer period in the biofilms. Indeed, biofilms of various microorganisms are known to be rich in protease activity [45]. Since GDST-RI peptides, as retro-inverso peptides, are expected to be more resistant to proteolytic degradation by host or bacterial proteases, this likely allows them to maintain activity longer in the biofilm environment, whereas L-peptides are degraded [46]. In 2015, de la Fuente-Núñez et al. showed that the RI variants of different literature peptides exhibited greater efficacy against biofilms than the original sequences against *K. pneumoniae* and *P. aeruginosa* [47]. Our data clearly support the notion that RI peptides offer real advantages against biofilms. This highlights a limitation of current ML systems, which are not designed to predict the activity of RI peptides. It would therefore be useful to train ML tools to also identify RI peptide sequences with potential anti-biofilm activity, provided that enough training data is available. This would contribute to increasing the treatment options for biofilm infections, which form two-thirds of all human infections [48].

Three-D HSEs are advanced, reproducible in vitro platforms that closely replicate the structure and function of native skin through their stratified epidermal layers, making them highly relevant for studying skin-pathogen interactions [49,50]. *S. aureus* is responsible for approximately half of all reported skin and soft tissue infections [51]. In our HSE infection model, the parent GDST-038 and GDST-045 peptides exhibited superior efficacy in killing both adherent and planktonic *S. aureus* compared with their RI variants and the reference peptide SAAP-148. In complex biological environments such as the skin, factors including peptide penetration, retention, and stability within infected tissue may account for the superior activity of the parent sequences. By utilizing a human skin cell-line-based, layered 3D HSE model, our study provides novel insights into peptide functionality in a physiologically relevant infection context.

In conclusion, peptides GDST-038 and GDST-045, initially selected through CalcAMP predictions and later complemented with their RI variants, exhibit broad-spectrum antimicrobial activity under physiological conditions, effectively kill MDR ESKAPE pathogens, target the bacterial membrane and cause very rapid bacterial death, show no resistance development, and demonstrate potent biofilm-killing activity. Moreover, their efficacy in the 3D HSE infection model further underlines their therapeutic potential. These features position them as promising alternatives to conventional anti-infective treatments, particularly for acute and biofilm-associated skin infections.

## 4. Materials and Methods

### 4.1. Peptides

#### 4.1.1. Study Strategy

The ML model CalcAMP [22] was used to identify novel AMP candidates. CalcAMP analyses key physicochemical and sequence-based features of peptide primary structures, extracting a distinct pattern to predict antimicrobial activity. Moreover, CalcAMP labels peptides as AMP or non-AMP based on experimentally determined activity thresholds from public databases. The set of peptides covered by the definitions and restrictions in patent WO2015088344 [52] served as the design space for guided selection of promising peptides based on predicted activity against Gram-negative and Gram-positive bacteria. The GDST peptides, derived from the human antimicrobial protein LL-37, follow the restrictions indicated in Table S3, with the 14-mer (KRLVKILKRWWRYL) as the starting point.

First, an in silico alanine scan of this 14-mer was performed to identify amino acids critical for antimicrobial activity (Table 1). Peptides were synthesized with screening purity and assessed in vitro for bactericidal activity, which appeared identical for all Ala-substitution variants. The Ala-scan thus indicated that the bactericidal activity of the peptide could not be attributed to any single amino acid side chain.

Subsequently, CalcAMP was used to predict antimicrobial activity for all 16,384 potential peptide sequences within the defined design space. These peptides were ranked based on predicted activity against Gram-negative bacteria, on a scale from 0 (minimal activity) to 1 (maximal activity). Since most peptides exhibited high predicted activity against Gram-positive bacteria, predictions against Gram-negative bacteria were prioritized for ranking. The top 14 sequences with the highest predicted activity were selected for further investigation (Table 2). Additionally, the six sequences predicted to have the lowest activity were included (Table S1).

#### 4.1.2. Peptide Synthesis

Peptides used in screening (Table 1, Table 2 and Table S1) were synthesized with C-terminal amidation and N-terminal acetylation using 9H-fluorenylmethyloxycarbonyl (Fmoc) chemistry in an automated peptide synthesizer (Syro II, MultiSyntech, Witten, Germany), as described by Hiemstra et al. [53]. Purification was performed with reverse-phase high-performance liquid chromatography (rpHPLC). Following initial screening, two lead candidates, their RI variants (peptides composed of D-amino acids in reversed order), and a set of well-characterized reference peptides from the literature (Table S2) were synthesized and purified as previously described [24], and used for further functional studies. Peptides were dissolved in phosphate-buffered saline (PBS; 140 mM NaCl, pH 7.4; Sigma-Aldrich, St. Louis, MO, USA) to create stock solutions of 2 mM.

### 4.2. Microorganisms and Culture

The bacterial strains used in this study included *S. aureus* JAR060131 [54] and the MDR ESKAPE pathogens *Enterococcus faecium* LUH15122, *S. aureus* LUH14616, *Klebsiella pneumoniae* LUH15104, *A. baumannii* RUH875 [55], *Pseudomonas aeruginosa* LUH15103, *Enterobacter cloacae* LUH15114, and the colistin-resistant *Escherichia coli* LUH15117. Before each experiment, bacteria from frozen stocks were cultured at 37 °C for 18–24 h on sheep blood agar plates (BioMérieux, Marcy-l’Étoile, France). All strains were then cultured from colonies either in tryptic soy broth (TSB; Oxoid, Basingstoke, UK) for *A. baumannii*, *S. aureus*, and *E. faecium*, or in brain heart infusion broth (BHI; Oxoid) for *K. pneumoniae*, *P. aeruginosa*, *E. cloacae*, and *E. coli* at 37 °C with shaking at 120 rpm for 18–24 h. Subsequently, bacteria were cultured to mid-logarithmic phase in the media described above at 37 °C with shaking at 120 rpm for 3 h. Bacteria were then washed twice with PBS and diluted to the required inoculum suspension concentration in Roswell Park Memorial Institute (RPMI)-1640 medium containing 20 mM HEPES and L-glutamine but without sodium bicarbonate (Sigma-Aldrich; hereafter referred to as RPMI), based on optical density at 620 nm. RPMI was selected because it supports bacterial growth without impairing AMP activity [56].

### 4.3. Bactericidal Activity

Bactericidal activity was assessed as described by de Breij et al. [57]. Briefly, mid-logarithmic phase bacterial cultures were diluted to a final concentration of 1 × 10^7^ colony-forming units (CFU)/mL in RPMI. Then, 10 µL was added to a final volume of 100 µL in RPMI (final concentration: 1 × 10^6^ CFU/mL). Cultures were exposed to peptides either in RPMI or RPMI supplemented with 50% (*v*/*v*) human plasma (hereafter referred to as 50% plasma), obtained from four healthy volunteers after informed consent, frozen after collection, and pooled (Sanquin, Amsterdam, The Netherlands). Assays were performed in duplicate using round-bottom polypropylene microtiter plates (Costar, Arlington, VA, USA) and repeated three times. In the initial screening phase (Table 1, Table 2 and Table S1), peptide solutions of 1, 10, and 100 μM (final concentration) were tested against *A. baumannii* RUH875 and *S. aureus* JAR060131 in RPMI and in 50% plasma. After this selection process, two lead candidates, their RI variants, and reference peptides were tested with a final concentration range of 0.12 to 120 µM (two-fold dilutions) in RPMI and in 50% plasma (Table 3). The peptides were also tested against ESKAPE pathogens, with Synthetic Antimicrobial and Antibiofilm Peptide (SAAP)-148 as a reference peptide (Table 4) [24].RPMI or 50% plasma served as no-treatment controls. After 2 and 18 h of incubation at 37 °C with shaking at 120 rpm in a humidified environment, the numbers of viable bacteria were quantified. Bactericidal activity was expressed as the lethal concentration 99.9% (LC99.9), defined as the lowest peptide concentration that killed ≥99.9% of bacteria within 2 h (LC99.9_2h_) or 18 h (LC99.9_18h_) compared to the initial inoculum.

### 4.4. Time–Kill Analysis

For time–kill analysis, a mid-logarithmic phase culture of *A. baumannii* RUH875 or *S. aureus* JAR060131 (final concentration: 1 × 10^6^ CFU/mL) was incubated in polypropylene tubes (Micronics, Chattanooga, TN, USA) with 0.5-, 1-, and 2-fold LC99.9_2h_ (RPMI) concentrations of GDST-038, GDST-045, their RI variants, or the reference peptide SAAP-148 in PBS, each in a total volume of 750 µL. Bacteria incubated in PBS alone served as no-treatment controls. At five time points (1, 2, 5, 30, and 120 min), duplicate 50 µL samples were taken and immediately mixed with 50 µL of 0.05% (*v*/*v*) sodium polyanethol sulfonate (SPS; Sigma-Aldrich) in PBS to neutralize residual peptide activity [58]. The number of viable bacteria was quantified by plating 10 µL aliquots of serial 10-fold dilutions onto sheep blood agar plates. Colonies were counted after 24 h of incubation at 37 °C.

### 4.5. SYTOX Green Assay

Membrane integrity of *A. baumannii* RUH875 and *S. aureus* JAR060131 (1 × 10^7^ CFU/mL) treated with GDST peptides and SAAP-148 was analysed using SYTOX Green (Invitrogen, Waltham, MA, USA), which fluoresces upon entering cells with permeabilized membranes and binding to their nucleic acids. Bacterial suspensions were prepared in RPMI containing 1 μM SYTOX Green and added to 96-well polypropylene flat-bottom plates (Greiner, Kremsmünster, Austria) containing 1/4×, 1/2×, 1×, and 2× LC99.9_2h_ concentrations of peptides (final volume: 100 μL). Control samples were exposed to RPMI only. As a positive control, heat-killed bacteria were incubated at 95 °C for 10 min to induce maximum membrane permeabilization. Fluorescence (excitation: 485 nm, emission: 520 nm) was monitored every minute for 90 min at 37 °C using a VANTAstar plate reader (Ortenberg, Germany), with peptides and positive controls added after the third cycle. Background fluorescence (SYTOX Green alone) was subtracted from all values. All assays were performed in triplicate and repeated twice independently.

### 4.6. Resistance Evolution

The potential for resistance development to GDST-038, GDST-045, and their RI variants was assessed as described by Habets and Brockhurst [59]. Resistance development to the antibiotics rifampicin and ciprofloxacin (both from Sigma-Aldrich) was evaluated for comparison. Briefly, 10 μL of a mid-logarithmic phase culture of *A. baumannii* RUH875 or *S. aureus* JAR060131 was added to 90 μL of peptide (final concentrations: 0.23 to 120 μM) or antibiotic (final concentrations: 0.03 to 256 μg/mL) solution in RPMI, in 96-well polypropylene round-bottom plates (Costar). All incubations were performed in quadruplicate. Plates were sealed with breathable seals (Greiner) and incubated at 37 °C with shaking at 120 rpm in a humidified environment for 18 h (*S. aureus*) or 48 h (*A. baumannii*).

The minimum inhibitory concentration (MIC), defined as the lowest concentration that inhibited visible bacterial growth, was then determined. Subsequently, 2 μL from the 0.5-fold MIC condition was transferred to fresh medium containing a concentration series ranging from 0.5- to 8-fold the new MIC. This procedure was repeated over 22 consecutive passages to monitor resistance development.

### 4.7. Biofilm Killing

Mid-logarithmic phase cultures of *A. baumannii* RUH875 or *S. aureus* JAR060131 were diluted to 1 × 10^8^ CFU/mL in RPMI supplemented with 1% glucose. A total of 100 μL of each suspension was added to flat-bottom polystyrene 96-well microplates (Costar) and incubated under static conditions at 37 °C in a humidified atmosphere for 24 h to allow biofilm formation. After 24 h, planktonic bacteria were removed, and wells were carefully washed with 100 μL PBS [60]. The biofilms were then exposed to 100 μL of GDST-038, GDST-045, their RI variants, or reference peptides at concentrations ranging from 1.88 to 120 μM in PBS. Control samples were exposed to 100 μL of PBS only.

Biofilms were treated at 37 °C for either 2 or 24 h. Peptide activity was neutralized by adding SPS (final concentration: 0.025% (*v*/*v*)) and incubating for 5 min at room temperature. Plates were sealed and sonicated for 5 min (Elmasonic, Singen, Germany; 45 kHz) at room temperature to dislodge adherent bacteria. This procedure does not affect bacterial viability [61]. Quantitative cultures were performed by plating serial 10-fold dilutions on sheep blood agar, and results were expressed as log_10_ CFU per well. The lower limit of detection was three CFU. Each condition was tested in six replicates.

### 4.8. Cytotoxicity Assessment

#### 4.8.1. Haemolytic Activity

Whole blood was collected from a healthy volunteer after informed consent, approved by the Institutional Review Board of AMC-UvA (BACON 1.8; approved 26 October 2018). Vacuette EDTA tubes were used (Greiner). To assess haemolytic activity, 180 μL of a freshly prepared 1% (*v*/*v*) human red blood cell suspension in PBS was mixed with 20 μL of peptide solution (0, 1, 10, or 100 μM in PBS). Controls included a zero-haemolysis blank (PBS alone) and a 100% haemolysis control (cells lysed by 1% Triton X-100 in PBS; Sigma-Aldrich). Samples were incubated at 37 °C for 45 min, then centrifuged at 2500× *g* rpm for 10 min. A total of 100 μL of supernatant was collected, and haemoglobin release was quantified by measuring absorbance at 540 nm using a UV−vis spectrophotometer (Synergy H1, Biotek, Winooski, VT, USA). A peptide was considered haemolytic if it caused >30% haemolysis relative to the 100% haemolysis control.

#### 4.8.2. Cytotoxicity for Human Skin Fibroblasts

BJ human skin fibroblast cells (ATCC CRL-2522; RRID: CVCL_3653; LGC Standards, Germany (September 2023)) were cultured in Dulbecco’s Modified Eagle Medium (DMEM; Gibco, Waltham, MA, USA) supplemented with 10% (*v*/*v*) foetal bovine serum (FBS; Sigma-Aldrich) and 1% (*v*/*v*) penicillin/streptomycin (pen/strep; Gibco). Cell suspensions (5 × 10^5^ cells/mL) were seeded into 96-well plates (100 μL/well; Thermo Scientific, Waltham, MA, USA) and allowed to adhere for 24 h. Monolayers were then exposed to two-fold serial dilutions of GDST peptides, their RI variants, and reference peptides (final concentrations: 0.05 to 60 µM) in fresh DMEM with 2% (*v*/*v*) FBS for 24 h at 37 °C in a humidified atmosphere with 5% CO_2_.

Cell viability and membrane integrity were assessed using the water-soluble tetrazolium salt (WST-1; Roche, Basel, Switzerland) assay and the lactate dehydrogenase (LDH; Abcam, Cambridge, UK) assay, respectively, according to manufacturer instructions. Results were expressed as percentages of metabolic activity and LDH leakage relative to untreated control cells and the positive lysis control, respectively. The cytotoxic concentration was defined as the lowest peptide concentration causing <70% metabolic activity and >30% LDH leakage. All experiments were performed in triplicate.

### 4.9. D Human Skin Equivalents

Human keratinocytes (Ker-CT cell line; ATCC CRL-4048; RRID: CVCL_S877; ATCC, Manassas, VA, USA (October 2017)) were used to generate human skin equivalents (HSEs) following the method described by Gent et al. [62]. Briefly, HSEs were cultured for 12 days, after which the culture medium was replaced with antibiotic-free medium, and cells were grown for an additional two days before infection (Figure S2). On the day of infection, HSEs were inoculated with 300 µL of *S. aureus* JAR060131 suspension (3 × 10^5^ CFU) and incubated for 1 h at 37 °C with 5% CO_2_. Following incubation, the bacterial suspension was removed, and HSEs were washed once with 500 µL PBS. HSEs were then treated for 4 h with PBS (control), GDST-038, GDST-045, their RI variants, or the reference peptide SAAP-148 at concentrations of 3.75, 15, and 60 μM, in triplicate. Subsequently, supernatants containing planktonic (non-adherent) bacteria were collected and kept on ice. Peptide activity was neutralized by adding SPS (final concentration: 0.025% (*v*/*v*)). Adherent bacteria within HSEs were quantified by homogenizing the tissues in 0.5 mL PBS containing 0.025% (*v*/*v*) SPS, using five zirconia beads (Ø 2 mm; BioSpec Products, Bartlesville, OK, USA) in a MagNA Lyser System (Roche) for three cycles of 30 sec at 7000 rpm, with 30 sec cooling intervals on ice. Both supernatants and homogenized fractions were serially diluted, plated on blood agar, and incubated overnight at 37 °C to determine CFU counts.

### 4.10. Statistics

All statistical analyses were performed using GraphPad Prism (version 10.2.0). Kruskal–Wallis and one-way ANOVA tests followed by Dunnett’s post hoc analysis were performed on log CFU counts in the biofilm killing and HSE infection models, respectively. For all analyses, *p*-values of ≤0.05 were considered statistically significant.

## Figures and Tables

**Table 1:** Alanine-scan analysis of peptide KRLVKILKRWWRYL, including predicted and experimentally determined bactericidal activity against *A. baumannii* and *S. aureus* at concentrations of 1, 10, and 100 µM in RPMI and in 50% plasma, alongside haemolytic activity (>30%). Residues shown in bold represent the amino acids replaced with alanine during the alanine-scan.

		Gram-Negative			Gram-Positive			Haemolysis
		Prediction	*A. baumannii*		Prediction	*S. aureus*		
Peptide	Sequence	Active	RPMI	50% Plasma	Active	RPMI	50% Plasma	>30%
GDST-021	**A**RLVKILKRWWRYL	0.758	10	10	0.858	≤1	100	100
GDST-022	K**A**LVKILKRWWRYL	0.753	≤1	10	0.851	10	100	100
GDST-023	KR**A**VKILKRWWRYL	0.775	10	≤1	0.890	≤1	100	100
GDST-024	KRL**A**KILKRWWRYL	0.761	≤1	10	0.891	10	100	100
GDST-025	KRLV**A**ILKRWWRYL	0.734	10	10	0.841	≤1	100	100
GDST-026	KRLVK**A**LKRWWRYL	0.800	≤1	10	0.878	≤1	100	100
GDST-027	KRLVKI**A**KRWWRYL	0.784	≤1	≤1	0.863	≤1	100	100
GDST-028	KRLVKIL**A**RWWRYL	0.763	≤1	10	0.863	≤1	100	100
GDST-029	KRLVKILK**A**WWRYL	0.776	≤1	10	0.859	≤1	100	100
GDST-030	KRLVKILKR**A**WRYL	0.781	10	≤1	0.849	≤1	10	100
GDST-031	KRLVKILKRW**A**RYL	0.773	≤1	≤1	0.825	10	100	100
GDST-032	KRLVKILKRWW**A**YL	0.702	≤1	10	0.806	≤1	100	100
GDST-033	KRLVKILKRWWR**A**L	0.837	≤1	10	0.788	≤1	>100	100
GDST-034	KRLVKILKRWWRY**A**	0.744	≤1	10	0.746	≤1	10	100

**Table 2:** Top 14 predicted active sequences derived from KR**L**VKI**L**K**RW**WR**Y**L (bold denotes amino acid substitutions permitted within the patent scope), each containing up to 5 amino acid substitutions. Experimentally assessed bactericidal activity against *A. baumannii* and *S. aureus* at concentrations of 1, 10, and 100 µM in RPMI and RPMI with 50% plasma, alongside haemolytic activity (>30%).

		**Gram-Negative**			**Gram-Positive**			**Haemolysis**
		**Prediction**	***A. baumannii***		**Prediction**	***S. aureus***		
**Peptide**	**Sequence**	**Active**	**RPMI**	**50% Plasma**	**Active**	**RPMI**	**50% Plasma**	**>30%**
GDST-035	KRWVKILKKAWRWL	0.912	10	10	0.965	≤1	10	100
GDST-036	KRWVKILKKWWRAL	0.909	≤1	10	0.838	≤1	100	100
GDST-037	KRWVKILKKAWRFL	0.907	10	≤1	0.959	≤1	100	100
GDST-038	KRWVKIAKKWWRLL	0.899	≤1	≤1	0.960	≤1	10	100
GDST-039	KRWVKILKRAWRWL	0.898	10	10	0.959	10	10	100
GDST-040	KRWVKILKKWWRLL	0.898	≤1	100	0.964	≤1	10	100
GDST-041	KRWVKILKRAWRFL	0.894	10	≤1	0.953	≤1	10	100
GDST-042	KRWVKILKKWWRFL	0.893	10	100	0.958	10	10	10
GDST-043	KRIVKILKKWWRFL	0.893	≤1	10	0.955	≤1	100	100
GDST-044	KRFVKILKKAWRWL	0.892	10	≤1	0.959	≤1	100	100
GDST-045	KRWVKILKKVWRFL	0.892	≤1	≤1	0.937	≤1	10	100
GDST-046	KRFVKILKKWWRLL	0.892	≤1	10	0.961	≤1	100	100
GDST-047	KRWVKIAKRWWRLL	0.891	10	≤1	0.951	≤1	100	100
GDST-048	KRIVKILKKWWRWL	0.890	10	10	0.963	10	100	100

**Table 3:** Bactericidal activity (LC99.9) and cytotoxicity assessment of GDST-038, GDST-045, their RI variants, and the reference peptides. Results are presented as medians (with ranges) from three independent experiments. Where no range is specified, the LC99.9 value was identical in the experiments.

	*A. baumannii* RUH875				*S. aureus* JAR060131				Cytotoxicity	
	RPMI		50% Plasma		RPMI		50% Plasma		LDH Leakage	Metabolic Activity
Peptide	2 h	18 h	2 h	18 h	2 h	18 h	2 h	18 h	>30%	<70%
GDST-038	0.94	0.47	0.94	0.47	0.94	0.94	1.88	0.94	3.75	3.75
	(0.47–0.94)		(0.47–0.94)	(0.47–0.94)		(0.47–0.94)		(0.47–0.94)		
GDST-038-RI	1.88	0.94	1.88	0.94	1.88	0.94	3.75	3.75	3.75	3.75
	(0.94–1.88)		(1.88–3.75)				(0.94–3.75)	(0.94–3.75)		
GDST-045	0.94	0.94	1.88	0.94	0.94	0.94	3.75	1.88	3.75	3.75
	(0.94–3.75)		(0.94–1.88)	(0.47–0.94)	(0.47–0.94)	(0.47–0.94)		(0.94–1.88)		
GDST-045-RI	1.88	0.94	1.88	0.94	1.88	0.94	3.75	1.88	3.75	3.75
			(1.88–3.75)	(0.94–1.88)		(3.75–7.5)	(0.94–1.88)			
LL-37	1.88	1.88	60	60	>120	>120	>120	>120	7.5	15
	(0.94–1.88)	(0.94–1.88)								
BP2	0.47	0.47	0.47	0.47	0.47	0.47	15	1.88	1.88	0.47
			(0.23–0.47)							
Pexiganan	0.23	0.47	0.23	0.47	0.47	0.23	7.50	0.94	1.88	0.94
		(0.23–0.47)			(0.47–0.94)			(0.94–1.88)		
SAAP-148	1.88	0.94	1.88	0.94	0.94	0.47	3.75	1.88	1.88	3.75
	(0.47–1.88)			(0.94–1.88)	(0.47–0.94)	(0.23–0.94)	(0.47–3.75)			
TC84	3.75	3.75	3.75	7.5	7.5	7.5	7.5	7.5	15	15
	(1.88–7.5)	(3.75–7.5)						(7.5–15)		

**Table 4:** Bactericidal activity (LC99.9) of GDST-038, GDST-045, their RI variants, and the reference peptide SAAP-148 against MDR ESKAPE pathogens. Results are presented as medians (with ranges) from three independent experiments. Where no range is specified, the LC99.9 value was consistent across experiments. “-” indicates not determined.

	GDST-038						GDST-038-RI					
	RPMI			50% Plasma			RPMI				50% Plasma	
Species	2 h	18 h		2 h	18 h		2 h		18 h		2 h	18 h
*E. faecium* LUH15122	1.88	0.94		1.88	7.5		3.75		0.94		1.88	3.75
		(0.94–1.88)										
*S. aureus* LUH14616	0.94	0.94		7.5	0.94		1.88		0.47		30	1.88
*K. pneumoniae* LUH15104	3.75	3.75		7.5	3.75		15		7.5		15	3.75
							(15–30)		(7.5–15)			
*A. baumannii* RUH875	0.94	0.47		0.94	0.47		1.88		0.94		1.88	0.94
	(0.47–0.94)			(0.47–0.94)	(0.47–0.94)		(0.94–1.88)				(1.88–3.75)	
*P. aeruginosa* LUH15103	7.5	3.75		15	15		15		7.5		30	15
	(7.5–15)	(3.75–15)							(3.75–7.5)			
*E. cloacae* LUH15114	3.75	1.88		60	1.88		30		1.88		60	1.88
									(1.88–3.75)			
*E. coli* LUH15117	0.94	1.88		-	-		3.75		0.94		-	-
	(0.94–1.88)	(0.94–1.88)							(0.94–1.88)			
	**GDST-045**				**GDST-045-RI**				**SAAP-148**			
	**RPMI**		**50% Plasma**		**RPMI**		**50% Plasma**		**RPMI**		**50% Plasma**	
**Species**	**2 h**	**18 h**	**2 h**	**18 h**	**2 h**	**18 h**	**2 h**	**18 h**	**2 h**	**18 h**	**2 h**	**18 h**
*E. faecium* LUH15122	0.94	0.94	0.94	3.75	0.94	0.94	1.88	3.75	0.47	0.47	30	3.75
					(0.94–1.88)	(0.47–0.94)			(0.47–0.94)			
*S. aureus* LUH14616	0.94	0.94	15	1.88	0.94	0.94	15	1.88	0.47	0.47	30	3.75
					(0.94–1.88)							
*K. pneumoniae* LUH15104	0.94	1.88	15	7.5	15	7.5	15	7.5	30	1.88	60	1.88
				(7.5–15)								(1.88–3.75)
*A. baumannii* RUH875	0.94	0.94	1.88	0.94	1.88	0.94	1.88	0.94	1.88	0.94	1.88	0.94
	(0.94–3.75)		(0.94–1.88)	(0.47–0.94)			(1.88–3.75)	(0.94–1.88)	(0.47–1.88)			(0.94–1.88)
*P. aeruginosa* LUH15103	1.88	3.75	60	30	3.75	3.75	30	30	0.47	1.88	30	30
		(3.75–7.5)			(3.75–7.5)							
*E. cloacae* LUH15114	1.88	1.88	60	30	15	3.75	120	30	>120	3.75	>120	0.94
						(3.75–7.55)						(0.94–1.88)
*E. coli* LUH15117	0.94	0.94	-	-	3.75	1.88	-	-	1.88	1.88	-	-
					(1.88–3.75)	(1.88–3.75)						

## Data Availability

The data that support the findings of this study are available from the corresponding author upon reasonable request.

## Supplementary Materials

The following supporting information can be downloaded at https://www.mdpi.com/article/10.3390/antibiotics14111172/s1. Figure S1: Cytotoxicity evaluation (cell viability and membrane integrity) of GDST-038, GDST-045, their RI variants, and reference peptides. Figure S2: Summary of the generation of 3D HSE using human keratinocytes (Ker-CT cells). Table S1: Peptide design space. Table S2: The six least-active predicted sequences. Table S3: Reference cationic AMPs used in this study.

## Abbreviations

The following abbreviations are used in this manuscript:

AMP(s)	Antimicrobial peptide(s)
AMR	Antimicrobial resistance
BHI	Brain heart infusion broth
BP2	Bactericidal peptide 2
CFU	Colony-forming unit
DMSO	Dimethyl sulfoxide
ESKAPE	*Enterococcus faecium*, *Staphylococcus aureus*, *Klebsiella pneumoniae*, *Acinetobacter baumannii*, *Pseudomonas aeruginosa*, *Enterobacter* spp.
Fmoc	9H-fluorenylmethyloxycarbonyl
GDST	Guided Designed Smart antimicrobial Therapeutic
HSE	Human skin equivalent
HK	Heat-killed
LDH	Lactate dehydrogenase
LC99.9	Lethal concentration killing 99.9% of bacteria
MDR	Multidrug resistant
MIC	Minimum inhibitory concentration
ML	Machine learning
PBS	Phosphate-buffered saline
RI	Retro-inverso
RPMI	Roswell Park Memorial Institute 1640 medium
SAAP-148	Synthetic antimicrobial and antibiofilm peptide 148
SPS	Sodium polyanethol sulfonate
TSB	Tryptic soy broth
WST-1	Water-soluble tetrazolium salt
