# Supplementary material for: Machine Learning-Identified Potent Antimicrobial Peptides Against Multidrug-Resistant Bacteria and Skin Infections
Source: Antibiotics (Basel). 2025 Nov 20;14(11):1172. doi: 10.3390/antibiotics14111172 (PMC12649225; doi:10.3390/antibiotics14111172)
Supplement: Supplementary file 1 [file antibiotics-14-01172-s001.zip › antibiotics-3925715-supplementary.pdf]

antibiotics

MDPI

Supplementary Materials

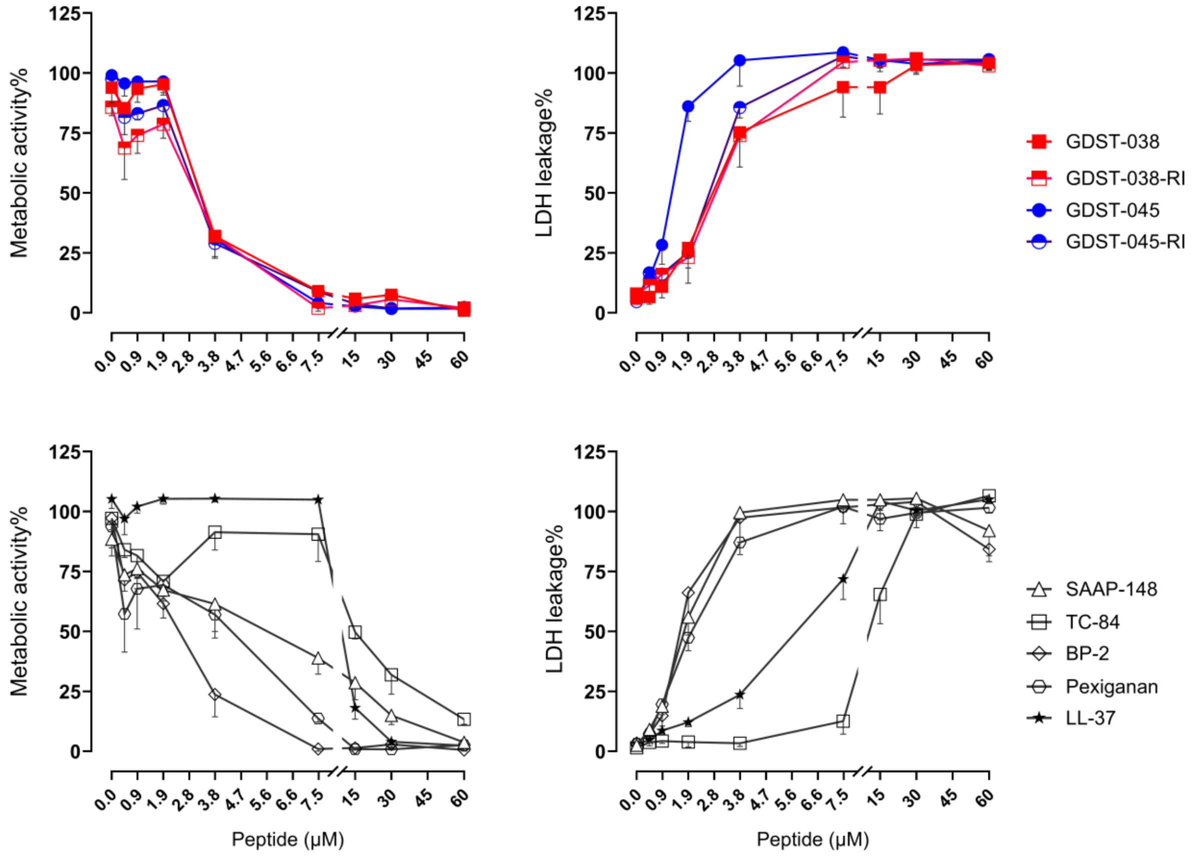

Figure S1. Cytotoxicity evaluation (cell viability and membrane integrity) of GDST-038, GDST-045, their RI variants, and reference peptides. Cell viability was assessed by measuring metabolic activity using a water-soluble tetrazolium salt conversion assay (WST-1; Roche). Loss of membrane integrity was assessed by the lactate dehydrogenase (LDH; Abcam) leakage assay, following the manufacturer's instructions. Results are expressed as a percentage relative to untreated and lysis controls. Cytotoxicity threshold are defined as the lowest peptide concentration causing <70% metabolic activity and the lowest concentration causing >30% LDH leakage. Experiments performed in triplicate.

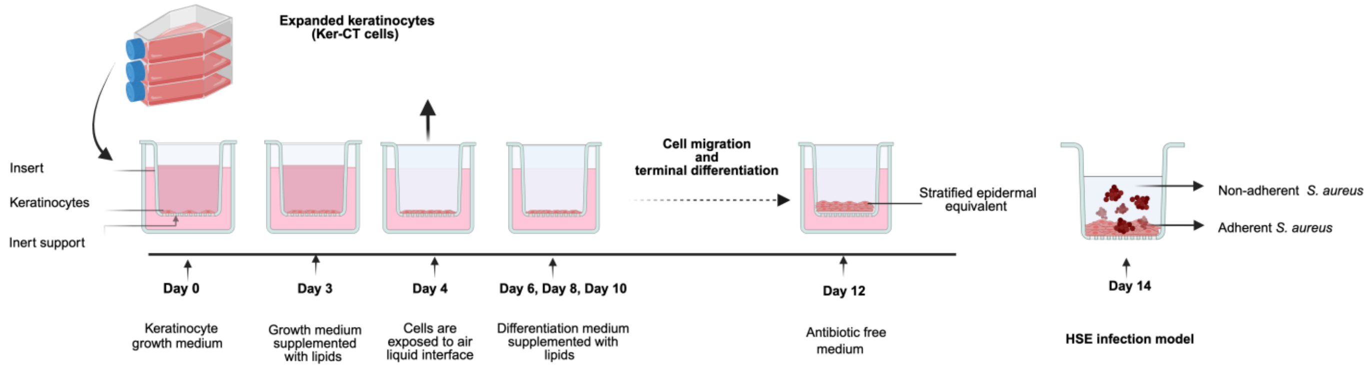

Figure S2. Summary of the generation of 3D HSE using human keratinocytes (Ker-CT cells). Cells were expanded in serum-free keratinocyte medium, seeded onto ThinCert transwells, and cultured in growth medium. Lipid mixtures were applied on days 3, 5, 7, and 9 to promote skin barrier formation. Air exposure on day 4 facilitated cell migration and terminal differentiation. On day 11, antibiotic-free medium was used to prepare for bacterial infection modelling. On day 14, HSEs were infected, allowing study of adherent and non-adherent bacteria. 3D HSE: 3-dimensional human skin equivalent.

Table S1. The six least-active predicted sequences, experimentally assessed for bactericidal activity against *A. baumannii* and *S. aureus* at concentrations of 1, 10, and 100 μM in RPMI and RPMI with 50% plasma, alongside haemolytic activity (>30%).

		**Gram-negative**			**Gram-positive**			**Haemolysis**
		Prediction	*A. baumannii*		Prediction	*S. aureus*		
**Peptide**	**Sequence**	Active	RPMI	50% plasma	Active	RPMI	50% plasma	>30%
GDST-052	KRVVKIQKHQWRQL	0.492	>100	>100	0.320	>100	>100	>100
GDST-053	AALVAILKRWWAYQ	0.264	100	>100	0.390	100	>100	>100
GDST-054	AHLVAILKRWWAYQ	0.269	100	>100	0.482	100	>100	>100
GDST-055	HALVAILKRWWAYQ	0.276	100	>100	0.470	100	>100	>100
GDST-056	AHLVAILKRWWHYQ	0.277	>100	>100	0.456	100	>100	>100
GDST-057	AALVHILKRWWHYQ	0.288	>100	>100	0.430	100	>100	>100

Table S2. Reference cationic AMPs used in this study.

**Peptide**	**Sequence**	**Clinical status**	**Charge (at pH=7.0)**	**Length**	**MW**	**Reference**
LL-37	LLGDFFRKSKEKIGKEFKRIVQRIKDFLRNLVPRTES	Preclinical	+6	37	4493	Johansson *et al.* - Journal of Biological Chemistry - 1998
BP2	GKWKLFKKAFKKFLKILAC	Preclinical	+7	19	2297	Kwakman *et al.* - Antimicrobial Agents and Chemotherapy - 2006
Pexiganan	GIGKFLKKAKKFGKAFVKILKK	Phase III	+9	22	2477	Ge *et al.* - Antimicrobial Agents and Chemotherapy - 1999
SAAP-148	LKRVWKRVFKLLKRYWRQLKKPVR	Preclinical	+11	24	3267	de Breij & Riool *et al.* - Science Translational Medicine - 2018
TC84	LRAMCIKWWSGKHPK	Preclinical	+4	15	1841	Omardien *et al.* - Biochimica et

	Biophysica Acta (BBA) - Biomembranes - 2018

Table S3. Peptide design space. [1]Core sequence of the design space: KRLVKILKRWWRYL; [2]Allowed amino acid substitutions, as defined in patent WO2015088344.

AA Position	1	2	3	4	5	6	7	8	9	10	11	12	13	14
**Sequence[1]**	**K**	**R**	**L**	**V**	**K**	**I**	**L**	**K**	**R**	**W**	**W**	**R**	**Y**	**L**
Allowed AA substitutions[2]	R	K	V	L	R	L	V	R	K	L	L	K	L	V
(per position)	A	A	F	F	A	V	F	A	A	V	V	A	V	F
	H	H	A	A	H	F	A	H	H	F	F	H	F	A
			I	I		A	I			A	A		A	I
			W	W		W	W			I	I		I	W
			Y	Y		Y	Y			Y	Y		W	Y
			Q	Q		Q	Q			Q	Q		Q	Q
